# Novel BODIPY-bridged cyclotriphosphazenes *Dedicated to Prof. Dr. Adem Kılıç on the occasion of his retirement.


**DOI:** 10.3906/kim-1907-44

**Published:** 2020-02-11

**Authors:** Hande ESERCİ, Ezel ÖZTÜRK, Elif OKUTAN

**Affiliations:** 1 Department of Chemistry, Faculty of Science, Gebze Technical University, Gebze, Kocaeli Turkey

**Keywords:** Boron-dipyrromethene, cyclotriphosphazene, photophysical, UV-Vis spectroscopy, photochemical

## Abstract

Three new 2-component unsubstituted ( **4P** ), diiodo- ( **5P** ), and dibromo- ( **6P** ) distyryl-BODIPY-bridged cyclotriphosphazene dimers were designed and synthesized. The newly synthesized BODIPY-cyclotriphosphazene systems were characterized by ^1^ H, ^13^ C, and ^31^ P NMR spectroscopy. The photophysical properties of the distryl-BODIPYs (4–6) and BODIPY-cyclotriphosphazene dyads ( **4P** – **6P** ) were studied by UV-Vis absorption and fluorescence emission spectroscopy. In these derivatives, the bino-type cyclotriphosphazene derivative bearing unsubstituted BODIPY unit **4P** exhibited high fluorescence and no singlet oxygen generation due to the lack of spin converter. The attachment of heavy atoms (iodine and bromine) enabled the production of singlet oxygen. The bino-type BODIPY-cyclotriphosphazenes ( **5P** and **6P** ) were also used as triplet photosensitizers in the photooxidation of 1,3-diphenylisobenzofuran to endoperoxide via generation of the singlet oxygen in dichloromethane. The singlet oxygen production of these compounds was also investigated via a direct method and produced a singlet oxygen phosphorescence peak at 1270 nm.

## 1. Introduction

Phosphazenes are one of the most studied classes of inorganic heterocyclic molecules [1]. The significance of this class is due to their wide application areas, such as medicine, biology, flame-retardant additives, membranes, microlithography, and ionic crystals [2–5]. In these studies, cyclotriphosphazenes are widely used as a core platform to develop tailor-made systems, because cyclotriphosphazene can easily be tuned by nucleophilic substitution reactions [6]. The readily available reactive phosphorus-chloride bonds provide an immense range of different molecular systems for targeted applications [7–9]. Moreover, phosphazenes possess thermal and chemical stabilities under different conditions, which is very important for specific applications areas [10,11]. Nucleophilic substitution reactions of phosphazenes with functional groups (alcohols, amines, thiols, etc.) have been widely studied [12–15]. Recently, derivatization of a phosphazene core with organic chromophores has attracted much attention. Specifically, 4, 4-difluoro-4-bora-3a, 4a-diaza-s-indacene BODIPY-cyclophosphazene systems were synthesized and characterized by several groups to investigate their photophysical and photochemical properties [16–18].

BODIPYs were proven to be versatile dyes with high molar absorption coefficients, large fluorescence quantum yields, and good solubility in various solvents [19]. A BODIPY core can be easily functionalized to tune the photophysical properties, such as the bathochromic shift of the UV-Vis absorbance via a Knoevenagel condensation reaction .[20]. However, the high fluorescent quantum yield of BODIPYs generally suggests trivial intersystem crossing, which hinders their role as triplet photosensitizers. To overcome this obstacle, brominated or iodinated BODIPY derivatives stand out by using the spin-orbital coupling effect of the heavy atom [19,21,22].

BODIPY-decorated cyclotriphosphazene derivatives have been readily reported as efficient singlet oxygen generators [23,24]. However, BODIPY-bridged cyclotriphosphazene derivatives, which can provide additional branching points, have never been reported. Herein, we reported the first examples of BODIPY-bridged cyclotriphosphazene dimers ( **4P** – **6P** ). Unsubstituted, iodinated, and brominated distyryl-BODIPY derivatives (4–6) bearing alkoxy functional groups were reacted with trimer and mono-BODIPY-bridged cyclotriphosphazene ( **4P** – **6P** ) derivatives were synthesized (Scheme). All of the prepared compounds were characterized by mass, ^31^ P, ^1^ H, and ^13^ C NMR spectroscopy. The photophysical properties of BODIPYs (4–7) and BODIPY-bridged cyclotriphosphazenes ( **4P** – **6P** ) were studied via UV-Vis and fluorescence emission techniques. Singlet oxygen generation abilities of BODIPY-bridged cyclotriphosphazenes ( **4P** – **6P** ) were investigated via both indirect and direct methods.

**Scheme:**
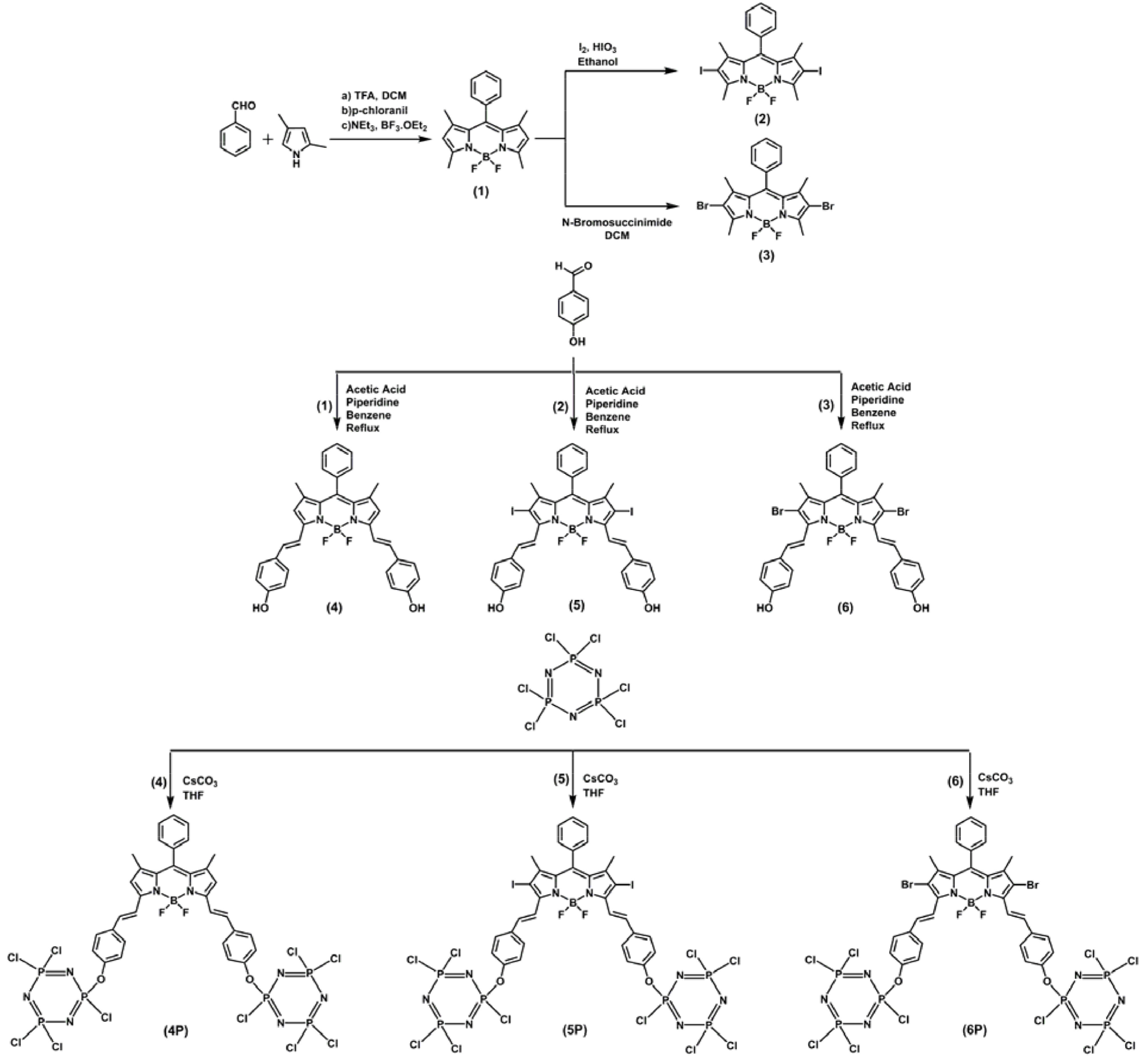
Synthesis of BODIPY-bridged cyclotriphosphazenes ( **4P** – **6P** ).

## 2. Experimental

### 2.1. Materials

Chloroform-d (CDCl _3_ ) , p-chloroanil, boron trifluoride diethyl etherate, silica gel, acetic acid, triethylamine, trifluoroacetic acid, and piperidine were obtained from Merck (Darmstadt, Germany). Subsequent chemicals were obtained from Sigma-Aldrich (St. Louis, MO, USA): ethanol, ethyl acetate, iodic acid, N-bromosuccinimide, dichloromethane, tetrahydrofuran 2,4-dimethyl pyrrole, and glacial acetic acid. 4-Hydroxy-benzaldehyde, benzene, and cesium carbonate were obtained from Alfa Aesar (Haverhill, MA, USA). The rest of the chemicals used in the synthesis were reagent grade unless otherwise specified.

### 2.2. Equipment

Absorption spectra of the compounds were inscribed with a Shimadzu 2101 UV spectrophotometer (Kyoto, Japan) in the UV-Vis region. Fluorescence excitation and emission spectra were obtained with a Varian Eclipse spectrofluorometer (Palo Alto, CA, USA) (1-cm path-length cuvette, RT). Singlet oxygen phosphorescence around 1270 nm was investigated using a Horiba Jobin-Yvon fluorometer (Kyoto, Japan) with a Hamamatsu NIR PMT 5509 (Hamamatsu, Japan) at –80 ◦ C. The fluorescence lifetime experiments were performed using a Horiba Jobin-Yvon-SPEX Fluorolog 3-2iHR instrument at excitation wavelengths with time-correlated singlephoton counting (TCSPC) module for signal acquisition. Mass spectra were obtained in linear modes on a Bruker Daltonics Microflex mass spectrometer (Billerica, MA, USA) equipped with a nitrogen UV-laser at 337 nm. The ^31^ P, ^1^ H, and ^13^ C NMR spectra were provided in solutions (CDCl _3_ ) with a Varian spectrometer (500 MHz). Analytical thin-layer chromatographies (TLC) were carried out on silica gel plates (Merck, Kieselgel 60 Å, 0.25-mm thickness with F254 indicator). Suction column chromatographies were made with silica gel (100–200 mesh).

### 2.3. Parameters for fluorescence quantum yields

The fluorescence quantum yields (Φ _F_ ) of compounds 4–6 and **4P** – **6P** were calculated by the relative procedure in Eq. (1) [25]:

(1)ΦF=ΦFStdFxAStdxn2FStdxAxnStd2

where the areas under the fluorescence emission curves of the compounds (4–6 and **4P** – **6P** ) and the standard are F and FStd , respectively. A and AStd represent the absorbances of compounds 4–6 and **4P** – **6P** , respectively. η values, as the refractive indices of the solvents, were considered in the determination of the fluorescence quantum yields in different solvents. Cresyl violet (Φ _F_ = 0.54/methanol) [26] was used as the standard.

### 2.4. Parameters for singlet oxygen

Singlet oxygen (Φ _Δ_ ) phosphorescence measurements were carried out using a Horiba Jobin-Yvon fluorometer with a Hamamatsu NIR PMT 5509. The intensity of singlet oxygen formation was obtained according to Eq.

(2)ΦΔ=ΦΔ(std)IΔ(compound)×A(std)IΔ(std)×A(compound)

Singlet oxygen quantum yields of compounds 4–6 and **4P** – **6P** were determined by employing DPBF as a trap molecule and methylene blue (MB) as a reference. Singlet oxygen generations were monitored by the decrease in absorbance of DPBF. A 630-nm (4.0 mW/cm ^2^ ) LED bulb was used as a light source and exposed from a cuvette distance of 5 cm, and absorbances were recorded at intervals for each irradiation (Eq. (3)):

(3)ΦΔ(compund)=ΦΔ(ref)[k(compound)k(ref)][F(ref)F(compound>][PF(ref)PF(compound)

Here, compound and ref designate the BODIPYs and BODIPY-cyclotriphosphazene (4–6 and **4P** – **6P** ) and MB, respectively. k is the slope of the change in maximum absorbance of DPBF (414 nm) with the irradiation time. F is the absorption correction factor (F = 1−10−OD , where OD is absorption at the irradiation wavelength), and PF is the light intensity (energy flux, mW/cm ^2^ ) .

### 2.5. Synthesis

Compounds 1–4 were synthesized with respect to the literature (Scheme) [27–29].

#### 2.5.1. Synthesis of compound 5

Compound 2 (100 mg, 0.173 mmol) and 4-hydroxybenzaldehyde (49 mg, 0.401 mmol) were dissolved in benzene (40 mL) under an argon atmosphere. Piperidine (0.3 mL) and glacial acetic acid (0.3 mL) were added to the solution and the reaction mixture was refluxed using a Dean-Stark apparatus until it was concentrated. The reaction was followed by TLC until the major product was a dark blue-colored compound. The reaction mixture was extracted from dichloromethane and water. Next, compound 5 was purified by silica gel column chromatography using dichloromethane and methanol (98:2) (yield: 73%).

Spectral data of 5: MS (MALDI-TOF) (DIT) m/z (%): calc. for C33 H25 BF2 I2 N2 O2 : 784.19; found: 784.15 [M+]. ^1^ H NMR (500 MHz, CDCl _3_ , 293 K, δ ppm): 8.00 (d, 3JH−H = 16.62 Hz, 2H, trans-CH), 7.41 (d, 3JH−H = 15.71, 2H, trans-CH), 7.40 (d, 3JH−H = 8.37, 6H, Ar-CH), 7.25 (br, 1H, Ar-CH), 7.17 (dd, 3JH−H = 6.29, 3JH−H = 6.30, 2H, Ar-CH) 6.75 (d, 3JH−H = 8.52 Hz, 4H, Ar-CH), 1.32 (s, 6H, -CH3) . ^13^ C NMR (126 MHz, CDCl _3_ , 293 K, δ ppm): 162.43, 154.48, 149.59, 143.46, 142.09, 139.18, 136.66, 133.28, 132.44, 132.29, 129.33, 115.75, 119.71, 29.53.

#### 2.5.2. Synthesis of compound 6

Compound 3 (100 mg, 0.207 mmol) and 4-hydroxybenzaldehyde derivative (64 mg, 0.518 mmol) were dissolved in benzene (40 mL) under an argon atmosphere. Piperidine (0.3 mL) and glacial acetic acid (0.3 mL) were added to the solution and the reaction mixture was refluxed using a Dean-Stark apparatus until it was concentrated. The reaction was followed by TLC until the major product was a dark blue-colored compound. The reaction mixture was extracted from dichloromethane and water. Compound 6 was purified by silica gel column chromatography using dichloromethane and methanol (97:3) (yield: 58%).

Spectral data of 6: MS (MALDI-TOF) (DHB) m/z (%): calc.: 690.19; found: 690.06 [M+]. ^1^ H NMR (500 MHz, CDCl _3_ , 293 K, δ ppm): 8.02 (d, 3JH−H = 16.6 Hz, 2H, trans-CH), 7.50 (d, 3JH−H = 16.9, 2H, trans-CH), 7.46 (m, 7H, Ar-CH), 7.22 (m, 2H, Ar-CH), 6.80 (d, 3JH−H = 8.2 Hz, 4H, Ar-CH), 1.33 (s, 6H, -CH3) . ^13^ C NMR (126 MHz, CDCl _3_ , 293 K, δ ppm): 158.47, 148.41, 140.85, 139.19, 134.80, 131.79, 129.39, 129.33, 129.27, 128.66, 128.31, 115.73, 115.19, 29.55, 25.40, 13.50.

#### 2.5.3. Synthesis of compound **4P**

A 100-mL round-bottomed flask was charged with tetrahydrofuran (50.0 mL) and purged with Ar for 15 min. Compound 4 (100 mg, 0.19 mmol) and cesium carbonate (159 mg, 0.49 mmol) were added to the reaction flask and stirred for 30 min. Trimer (163.0 mg, 0.47 mmol) was poured into the reaction mixture and the reaction mixture was stirred for 16 h at room temperature. The precipitated salt (CsCl) was filtered off and the solvent was removed at reduced pressure. The resulting white solid was subjected to column chromatography using n-hexane and ethyl acetate (3:2) as the mobile phase (yield: 55%).

Spectral data of **4P** : MS (MALDI-TOF) (NOM) m/z (%): calc.: 1154.77; found: 1154.18 [M+]. ^31^ P NMR (202 MHz, CDCl _3_ , δ ppm): 22.47 (d, 2JP−P = 60.59 Hz, 2P, PCl2) , 12.12 (t, 2JP−P = 60.59 Hz, 1P, PClOPh) spin system: A2 X. ^1^ H NMR (500 MHz, CDCl _3_ , 293 K, δ ppm): 7.72 (d, 3JH−H =16.5 Hz, 2H, trans-CH), 7.68 (d, 3JH−H =7.8 Hz, 4H, Ar-CH), 7.53 (m, 3H, Ar-CH), 7.34 (m, 6H, Ar-CH), 7.24 (d, 3JH−H =16.9 H, 2H, trans-CH), 6.66 (s, 2H, -CH), 1.48 (s, 6H, -CH3) . ^13^ C NMR (126 MHz, CDCl _3_ , 293 K, δ ppm): 152.27, 149.51, 149.41, 142.60, 139.72, 135.26, 134.92, 134.44, 133.59, 129.21, 128.94, 128.29, 121.78, 121.73, 120.12, 117.93, 25.62.

#### 2.5.4. Synthesis of compound **5P**

A 100-mL round-bottomed flask was charged with tetrahydrofuran (50.0 mL) and purged with Ar for 15 min. Compound 5 (150 mg, 0.191 mmol) and cesium carbonate (149 mg, 0.459 mmol) were added to the reaction flask and stirred for 30 min. Trimer (132.7 mg, 0.382 mmol) was poured into the reaction mixture and stirred for 16 h at room temperature. The precipitated salt (CsCl) was filtered off and the solvent was removed at reduced pressure. The resulting green solid was subjected to column chromatography using n-hexane and ethyl acetate (3:1) as the mobile phase (yield: 40%).

Spectral data of **5P** : MS (MALDI-TOF) (DIT) m/z (%): calc. for C33 H23 BCl10 F2 I2 N8 O2 P6 : 1406.56; found: 1406.58 [M+]. ^31^ P NMR (202 MHz, CDCl _3_ , δ ppm): 22.49 (d, 2JP−P = 61.08 Hz, 2P, PCl2) , 11.93 (t, 2JP−P = 61.08 Hz, 1P, PClOPh) spin system: A2 X. ^1^ H NMR (500 MHz, CDCl _3_ , 293 K, δ ppm): 8.06 (d, 3JH−H = 16.58 Hz, 2H, trans-CH), 7.52 (d, 3JH−H = 8.06 Hz, 2H, Ar-CH), 7.49 (d, 3JH−H = 16.57 Hz, 2H, trans-CH), 7.44 (br, 1H, Ar-CH), 7.40–7.37 (m, 4H, Ar-CH), 7.17–7.12 (m, 6H, Ar-CH) 1.43 (s, 6H, -CH3) . ^13^ C NMR (126 MHz, CDCl _3_ , 293 K, δ ppm): 147.52, 141.84, 107.64, 104.96, 104.93, 66.93, 34.54, 31.99, 29.09, 25.38, 23.71, 23.17.

#### 2.5.5. Synthesis of compound **6P**

A 100-mL round-bottomed flask was charged with tetrahydrofuran (50.0 mL) and purged with Ar for 15 min. Compound 6 (30 mg, 0.04 mmol) and cesium carbonate (34 mg, 0.1 mmol) were added to the reaction flask and stirred for 30 min. Trimer (30.2 mg, 0.08 mmol) was poured into the reaction mixture and stirred for 16 h at room temperature. The precipitated salt (CsCl) was filtered off and the solvent was removed at reduced pressure. The resulting green solid was subjected to column chromatography using n-hexane and tetrahydrofuran (4:1) as the mobile phase (yield: 30%).

Spectral data of **6P** : MS (MALDI-TOF) (DIT) m/z (%): calc.: 1312.56; found: 1312.091 [M+]. ^31^ P NMR (202 MHz, CDCl _3_ , δ ppm): 22.5 (d, 2JP−P = 60.88 Hz, 2P, PCl2) , 11.96 (t, 2JP−P = 60.83 Hz, 1P, PClOPh) spin system: A2 X. ^1^ H NMR (500 MHz, CDCl _3_ , 293 K, δ ppm): 8.07 (d, 3JH−H = 17. ^1^ Hz, 2H, trans-CH), 7.67 (d, 3JH−H = 8.6, 4H, Ar-CH), 7.62 (d, 3JH−H = 17.2 Hz, 2H, trans-CH), 7.53 (m, 3H, Ar-CH), 7.30 (d, 3JH−H = 8.7 Hz, 6H, Ar-CH), 1.41 (s, 6H, -CH3) . ^13^ C NMR (126 MHz, CDCl _3_ , 293 K, δ ppm): 129.51, 129.50, 129.15, 129.12, 128.06, 121.74, 121.70, 118.79, 110.56, 67.93, 53.36, 25.49, 13.69.

## 3. Results and discussion

### 3.1. Synthesis and structural characterization

The synthetic pathways to prepare the BODIPYs (4–6) and BODIPY-bridged cyclotriphosphazenes ( **4P** – **6P** ) in this study are shown in the Scheme. Compounds 1–3 were synthesized according to methods in the literature [27–29]. The synthetic strategy to prepare distyryl-BODIPYs (4–6) bearing the -OH functional group was also based on methods in the literature and initially included the reactions of BODIPYs 1–3 with 4- hydroxybenzaldehyde in benzene using a Dean-Stark apparatus via Knoevenagel condensation conditions [27]. BODIPYs (4–6) in tetrahydrofuran solutions were then reacted with an excess of hexachlorocyclotriphosphazene (trimer) in the presence of Cs2 CO3 to prepare BODIPY-bridged cyclotriphosphazenes ( **4P** – **6P** ) from nucleophilic displacement reactions. The progress of the reactions was followed by TLC and the products were purified by silica-gel column chromatography with proper eluent systems (see Section 2). Identifications of the BODIPYs (1–6) and BODIPY-cyclotriphosphazenes ( **4P** – **6P** ) were performed using ^31^ P, ^1^ H, and ^13^ C NMR spectroscopy and mass spectrometry. The results confirmed the established formulations, where the protondecoupled ^31^ P NMR spectra of BODIPY-bridged cyclotriphosphazenes **4P** – **6P** displayed the expected AX2 spin systems with 2 sets of signals corresponding to the P-OPhCl groups at ~11.9–12.1 ppm and the PCl2 groups at ~22.5 ppm.

### 3.2. Photophysical properties

**Figure 1:**
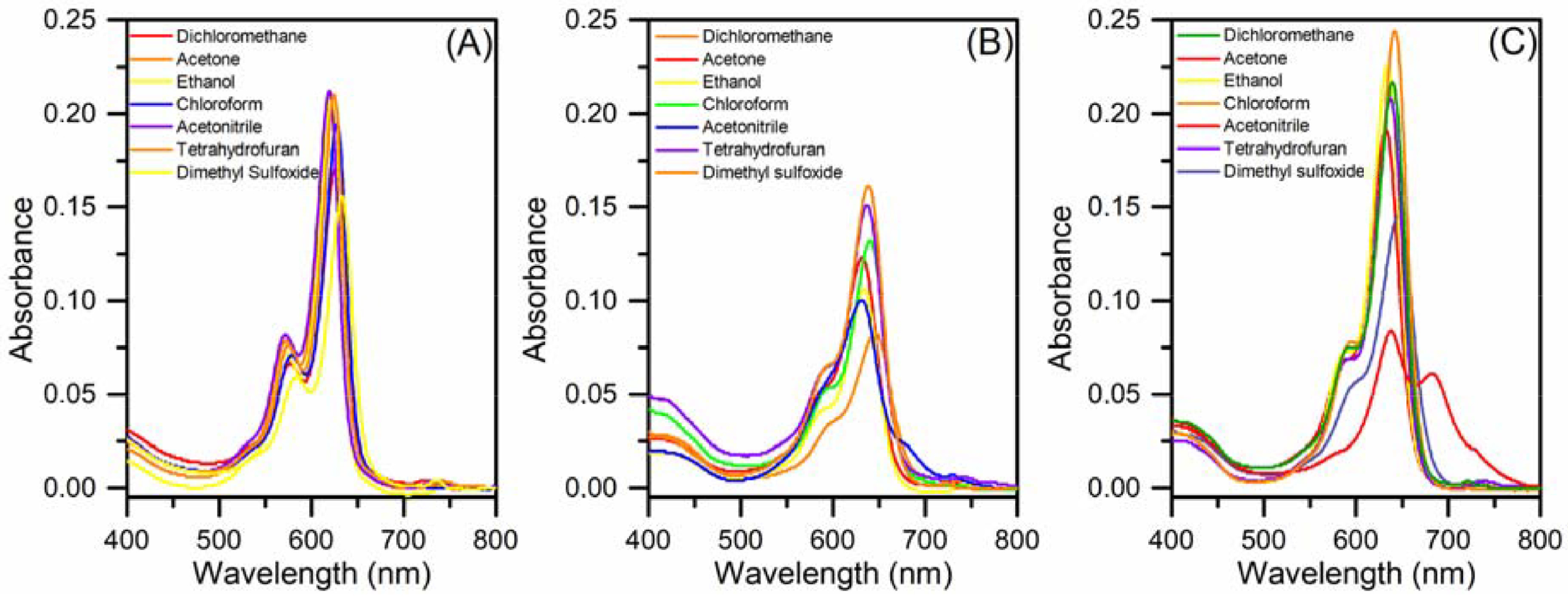
Absorption spectra of compounds (A) **4P** , (B) **5P** , and (C) **6P** in various solvents.

**Figure 2:**
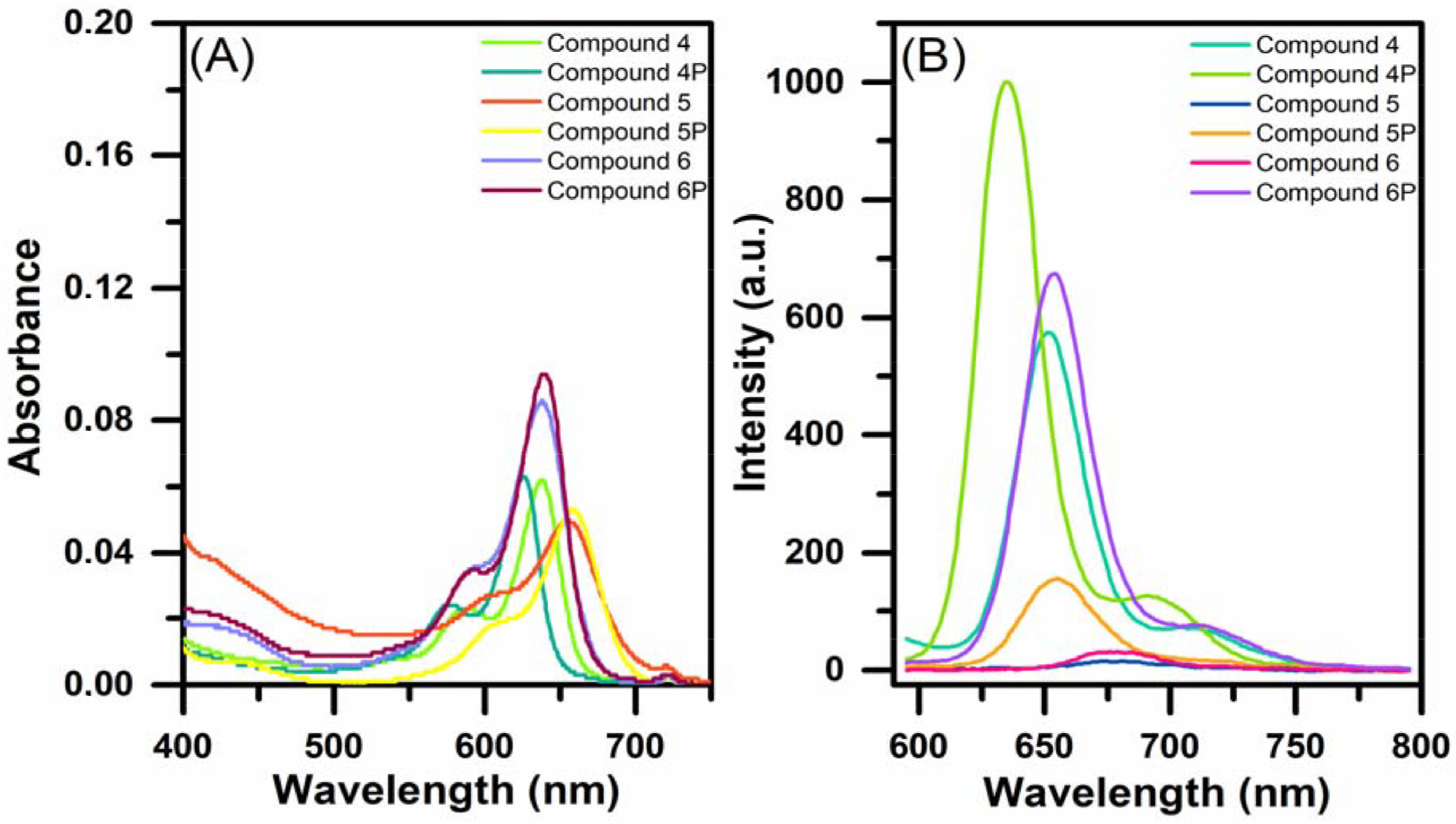
(A) UV-Vis absorption (2 μM) and (B) fluorescence emission (1 μM, λex = 580 nm) features of compounds 4–6 and **4P** – **6P** .

**Figure 3:**
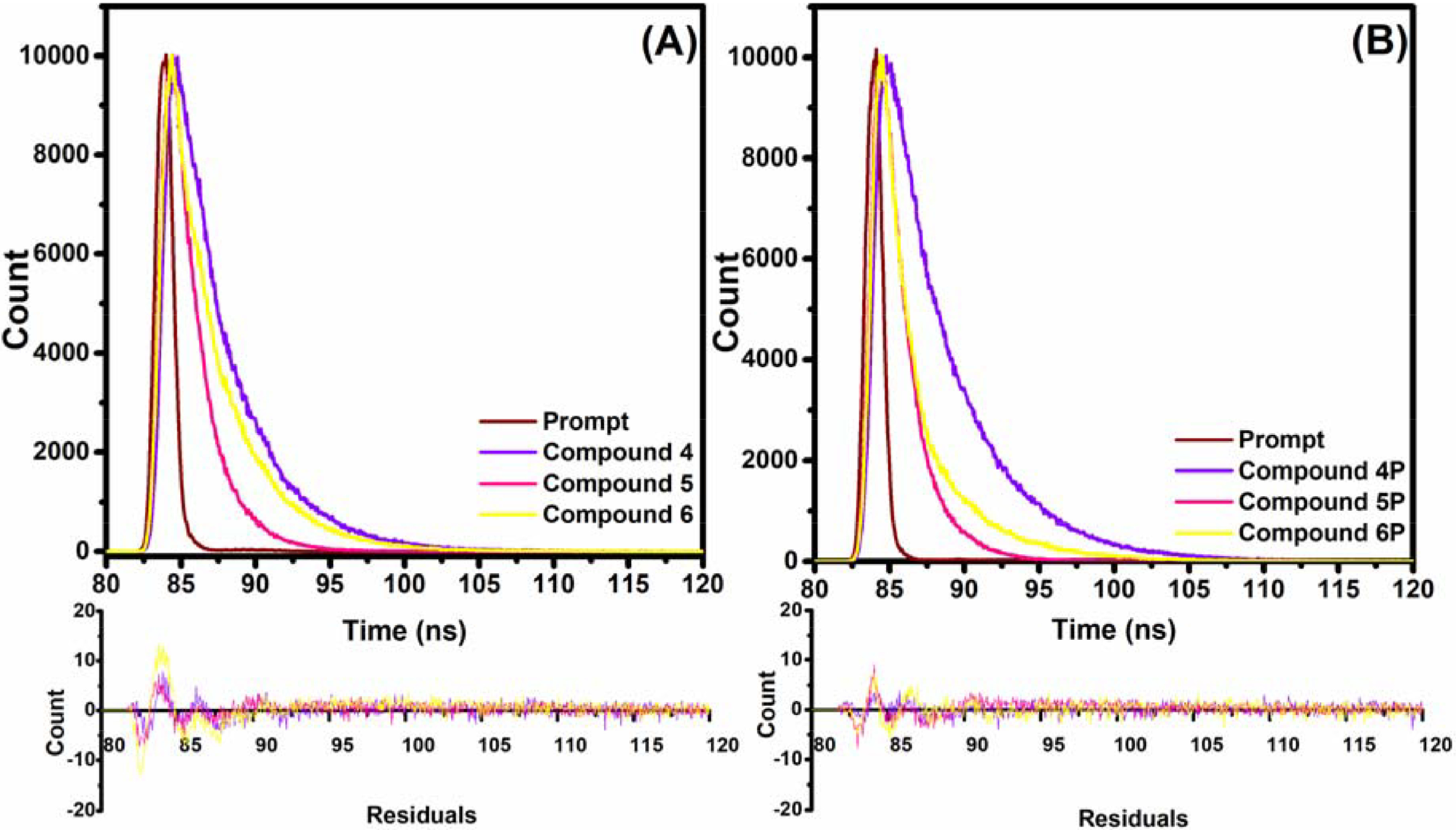
Fluorescence decay profiles of compounds (A) 4–6 and (B) **4P** – **6P** using a laser excitation source of 670 nm.

The electronic UV-Vis absorption spectra of BODIPY-cyclotriphosphazenes **4P** – **6P** were recorded in different solvents (Figure 1). The absorption profile of compound **4P** was observed to be similar in all of the studied solvents (dichloromethane, acetone, ethanol, chloroform, acetonitrile, tetrahydrofuran, and dimethyl sulfoxide) with maximum absorptions at ~622 nm. I2 -BODIPY-cyclotriphosphazene **5P** exhibited parallel absorption peaks with different intensities, where the maximum absorption intensity observed was in dichloromethane at 638 nm and the lowest was in dimethyl sulfoxide with a slight (~8 nm) bathochromic shift. Compound **6P** was influenced by the solvent and exhibited the most intense absorbances in chloroform, ethanol, and dichloromethane at 640 nm, whereas the lowest absorption was displayed in acetonitrile with an additional peak at ∼683, while the other selected solvent intensities were in the middle. Because all three compounds ( **4P** – **6P** ) displayed optimum absorption characteristics, dichloromethane was selected as the solvent to be studied. The concentrations of the compounds were fixed at 2 μM and measured at room temperature. Next, the molar extinction coefficients (ε) of BODIPYs 4–6 and BODIPY-cyclotriphosphazene derivatives **4P** – **6P** were determined for compounds 4–6 as 7.18, 5.22, and 6.44 M−1 × cm−1 and for compounds **4P** – **6P** as 13.74, 7.87, and 10.40 M−1 × cm−1 , respectively, in dichloromethane. The photophysical data are summarized in the Table. The electronic absorption characters of the distyryl-BODIPYs (4–6) and BODIPY-bridged cyclotriphosphazene derivatives ( **4P** – **6P** ) were studied in dichloromethane (Figure 2A). Maximum absorbance wavelengths of compounds 4–6 were determined as 638, 655, and 638 nm, which is characteristic for distyryl- BODIPY derivatives and caused by S0 –S1 transitions [19]. These characteristic transition bands were also observed for compounds **4P** – **6P** at 625, 658, and 639 nm, respectively. The absorption bands of the BODIPYbridged cyclotriphosphazenes were observed to be blue-shifted when compared to the parent BODIPY derivatives (4–6). Fluorescence emission spectra of the BODIPYs (4–6) and BODIPY-cyclotriphosphazenes ( **4P** – **6P** ) upon excitation at 580 nm are presented in Figure 2B. BODIPY derivative 4 exhibited strong emission at 634 nm with an excitation wavelength at 580 nm in dichloromethane. The fluorescence emission of diiodo- and dibromo- BODIPY derivatives 5 and 6 was ∼678 nm, whereby the fluorescence intensity of dibromo-BODIPY 6 stayed between that of 4 and 5, as expected [30]. The maximum fluorescence emissions of the BODIPY-bridged cyclotriphosphazenes ( **4P** – **6P** ) were at 634, 655, and 653 nm with hypsochromic shifts of 17–25 nm compared to those of related BODIPY derivative fluorescence with a similar but slightly broader spectral shape. The fluorescence quantum yields were calculated via the relative method using cresyl violet as the reference (Φref = 0.54/methanol) [26] and quantified as 0.32, 0.01, and 0.01 for compounds 4–6 and 0.25, 0.05, and 0.14 for **4P** – **6P** , respectively (Table). Moreover, the fluorescence lifetimes were obtained using TCSPC and the results are depicted in Figure 3 and the Table.

**Table:** Photophysical and photochemical features of compounds 4–6 and **4P** – **6P** .

Compound	Absorption wavelength λab (nm)	Emission Êwavelength λem (nm)	ΔStokes (nm)	∗ε, 104 (M−1 cm−1)	**τF (ns)	***Φ _F_	†Φ _Δ_
4	586, 638	651, 711	13	7.18	3.57	0.32	-
5	601, 655	677	21	5.22	1.78	0.01	-
6	591, 638	678	21	6.44	3.22	0.01	-
**4P**	576, 625	634, 693	10	13.74	4.33	0.25	-
**5P**	609, 658	655	16	7.87	1.68	0.05	0.24
**6P**	591, 639	653, 712	14	10.40	4.03	0.14	0.89

*Molar extinction coefficients, **fluorescence lifetime, ***fluorescence quantum yield, †singlet oxygen quantum yield via chemical method.

### 3.3. Photochemical properties

**Figure 4:**
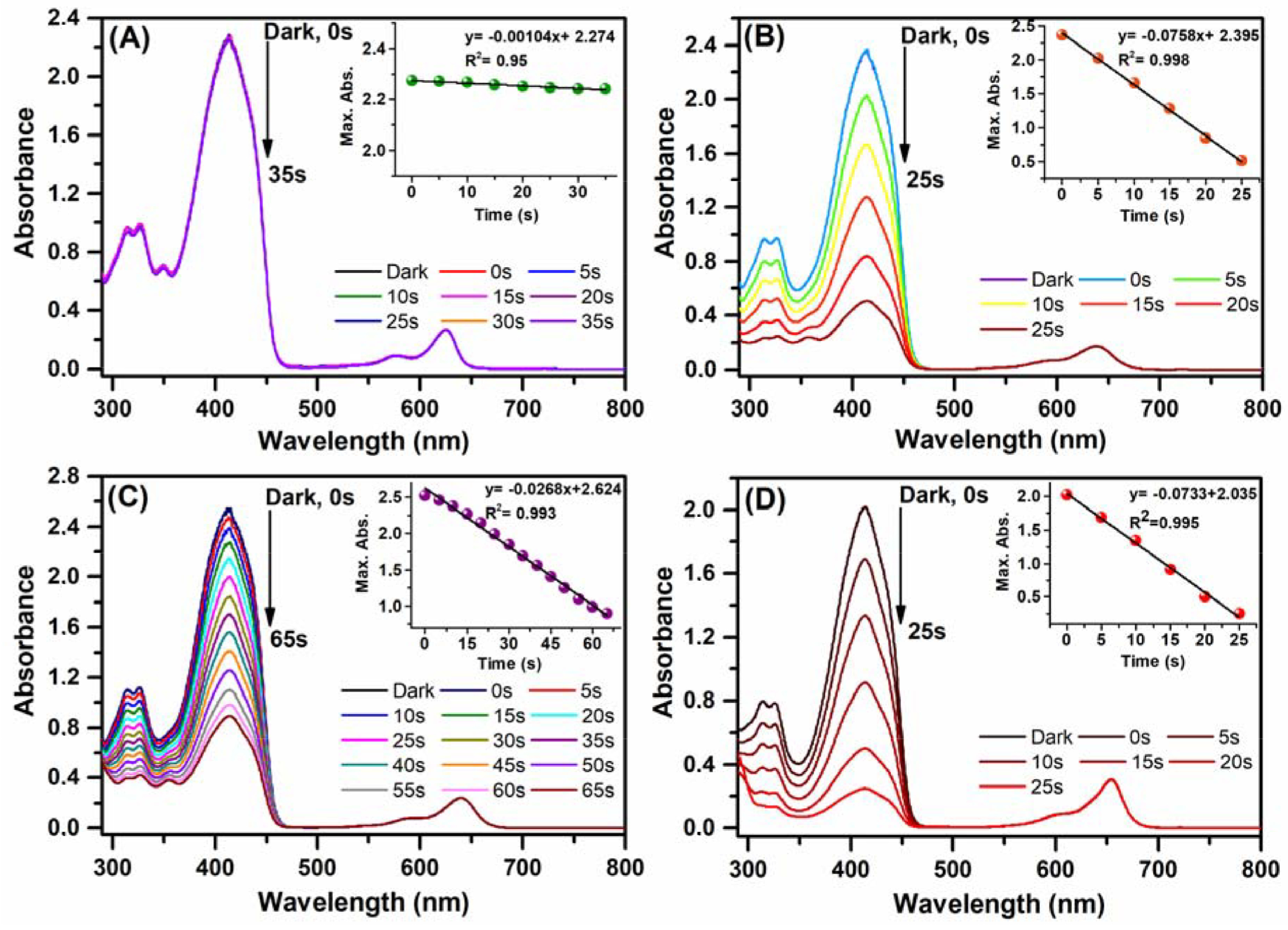
Absorbance spectra of DPBF after each irradiation in the presence of compounds (A) **4P** , (B) **5P** , (C) **6P** , and (D) MB in dichloromethane (2 μM).

**Figure 5:**
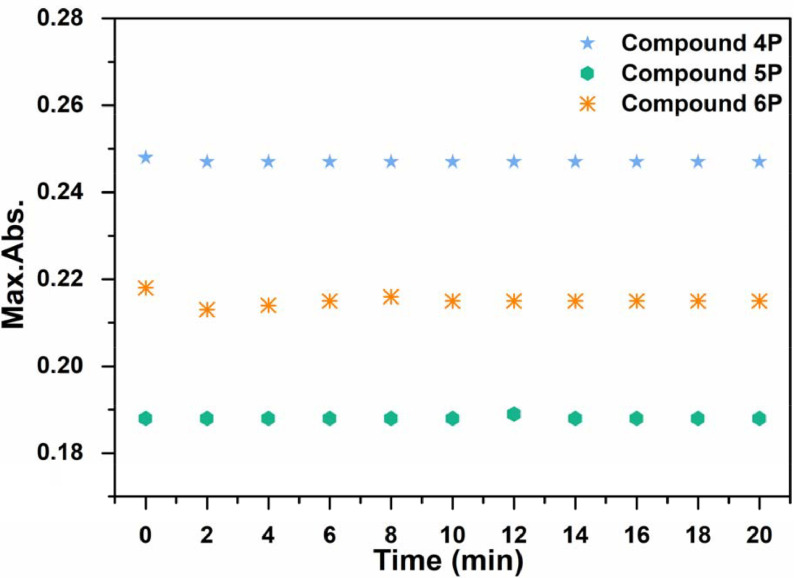
The photostabilities of BODIPY-cyclotriphosphazene conjugates ( **4P** – **6P** ).

Photosensitizers can be described as chemical tools that generate reactive oxygen species (ROS) via light illumination [31]. As one of the ROS, singlet oxygen is produced through energy delivery from a photosensitizer’s triplet energy state to triplet oxygen (ground state molecular oxygen, 3 O2) [32]. A fashionable strategy to enhance the efficiency of the triplet energy state for BODIPY chemistry is the covalent attachment of iodine or bromine to the 2 and 6 positions of the BODIPY core [30]. Herein, singlet oxygen production of the unsubstituted ( **4P** ), diiodo- ( **5P** ), and dibromo- ( **6P** ) substituted BODIPY-bridged cyclotriphosphazene systems was studied. The singlet oxygen formation abilities of the systems were first identified by chemical method via pursuing the photooxidation of DPBF [6]. The 2 μM dichloromethane solutions of the BODIPY-cyclotriphosphazenes ( **4P** – **6P** ) and DPBF (absorbance set to ∼2.3 ± 0.1) were first kept in the dark for 15 min, to eliminate possible side reactions, and then the solutions were irradiated for 5 s until the DPBF (414 nm) absorption was terminated with red LED (630 nm) systematically. Reductions in the absorption bands of DPBF were monitored to enable calculation of singlet oxygen generation yields via the indirect method (Figure 4). As expected, the absorbance of DPBF as a trap molecule (414 nm) did not display any significant alteration upon irradiation of compound **4P** due to the lack of a heavy atom effect, as expected (Figure 4A). In the case of compounds **5P** and **6P** , a heavy atom effect (iodine and bromine) was asserted and as soon as irradiation was initiated, about 80% and 14% of the DPBF absorptions vanished in 25 s (Figures 4B and 4C). Moreover, the photooxidation experiment of DPBF was performed with MB as the standard (Φ _Δ_ = 0.52 in dichloromethane) under the same experimental conditions (Figure 4D). Data for the compounds were plotted as the maximum absorption of the trap molecule versus the irradiation time to obtain the slopes of the graphics. The singlet oxygen quantum yields were determined via a relative method using MB as the standard, according to Eq. (3) (see Section 2). The singlet oxygen quantum yields of compounds **5P** and **6P** were determined as 0.89 and 0.24, respectively. The iodinated BODIPY-cyclotriphosphazene system **5P** exhibited more singlet oxygen yield than MB and compound **6P** . In addition, the photostabilities of the BODIPY-cyclotriphosphazenes ( **4P** – **6P** ) were studied since photostability upon excitation is an expected behavior [6]. Compounds **4P** – **6P** were excited with the same LED used in the photooxidation studies of DPBF for 20 min and no significant alteration occurred in the absorptions (Figure 5).

**Figure 6:**
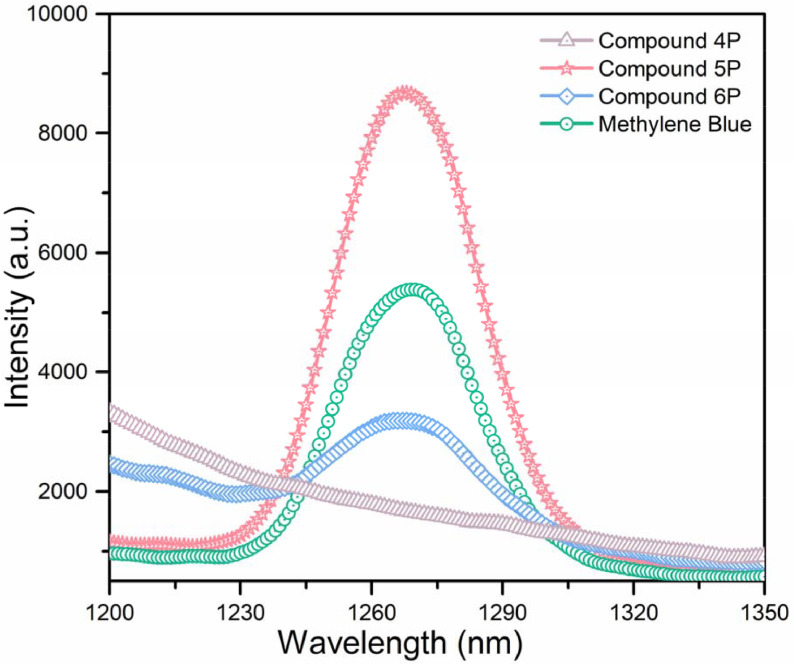
Singlet oxygen phosphorescence with sensitization of compounds **4P** – **6P** and MB in dichloromethane (maximum absorptions were set to 0.2 for all compounds).

The singlet oxygen generation characters were then examined by measuring singlet oxygen signature phosphorescence at 1270 nm for dyads ( **4P** – **6P** ). Compounds **4P** – **6P** and MB were excited at their excitation wavelengths with a xenon-arc source and detected with a near-IR sensitive detector. Equal absorptivities (0.2) for all of the compounds and MB in dichloromethane were excited and the BODIPY-bridged cyclotriphosphazene derivative bearing iodine substituents ( **5P** ) gave the strongest phosphorescence at 1270 nm, which was consistent with the photochemical singlet oxygen experiments. MB and compound **6P** also displayed phosphorescence peaks with reduced intensity (Figure 6).

### 3.4. Conclusions

Three novel BODIPY-bridged cyclotriphosphazenes composed of 2 cyclotriphosphazene and unsubstituted-( **4P** ), iodinated- ( **5P** ), and brominated- ( **6P** ) distyryl BODIPY subunits were designed and prepared via nucleophilic substitution reaction. Photophysical properties, such as UV-Vis absorption, fluorescence emission, fluorescence quantum yield, and lifetime of the parent BODIPY derivatives (4–6) and BODIPY-cyclotriphosphazenes ( **4P** – **6P** ) were studied. Absorption bands of the BODIPY-bridged cyclotriphosphazenes ( **4P** – **6P** ) were found to have shifted to blue relative to the parent BODIPY derivatives. The singlet oxygen generation efficiencies and photostabilities of compounds **4P** – **6P** were investigated by both direct and indirect (chemical) methods by comparing them with MB as the standard. The dimer, containing unsubstituted BODIPY-cyclotriphosphazene **4P** , exhibited no singlet oxygen generation, as expected, while the heavy atom-substituted iodine ( **5P** ) and bromine ( **6P** ) were found to have generated singlet oxygen with both methods. Iodination of BODIPY was found to be more effective than bromination to generate triplet photosensitization, and the singlet oxygen quantum yields were calculated with the indirect method and found as 0.89 and 0.24 for **5P** and **6P** , respectively. We suggest that BODIPY-bridged cyclotriphosphazenes exhibit excellent capacity as photosensitizers and can be used for the development of novel systems.
